# Distress Analysis of Mice with Cervical Arteriovenous Fistulas

**DOI:** 10.3390/ani11113051

**Published:** 2021-10-26

**Authors:** Wentao Xie, Rupert Palme, Clemens Schafmayer, Dietmar Zechner, Brigitte Vollmar, Eberhard Grambow

**Affiliations:** 1 Rudolf-Zenker-Institute for Experimental Surgery, University Medical Center Rostock, 18057 Rostock, Germany; dietmar.zechner@uni-rostock.de (D.Z.); brigitte.vollmar@med.uni-rostock.de (B.V.); eberhard.grambow@med.uni-rostock.de (E.G.); 2 Department of Vascular and Thyroid Surgery, Department of General Surgery, The First Affiliated Hospital of Anhui Medical University, Hefei 230022, China; 3 Unit of Physiology, Pathophysiology and Experimental Endocrinology, Department of Biomedical Sciences, University of Veterinary Medicine Vienna, 1210 Vienna, Austria; Rupert.Palme@vetmeduni.ac.at; 4 Department for General, Visceral, Thoracic, Vascular and Transplantation Surgery, University Medical Center Rostock, 18057 Rostock, Germany; clemens.schafmayer@med.uni-rostock.de

**Keywords:** hemodialysis access, animal model, distress, animal welfare, 3R rule

## Abstract

**Simple Summary:**

Functional hemodialysis access is essential for the survival of patients with end-stage renal disease. Although various guidelines recommend autologous arteriovenous fistula as the first choice for hemodialysis, it is still the Achilles heel for patients. Several in vivo models have been used to study and improve the mechanisms of vascular remodeling of arteriovenous fistula. However, some models have the disadvantage of having anatomical features or a hemodynamic profile different from that of the arteriovenous fistula in humans. In the presented cervical arteriovenous fistula model, these disadvantages were eliminated. It resembles the human physiology and is an ideal animal model for arteriovenous fistula research. Moreover, in order to understand the impact of this model on animal welfare, the distress of this new animal model was analyzed. Body weight, faecal corticosterone metabolites, burrowing activity, nesting behaviour and distress scores were analysed after fistula creation and during the following three weeks. The physiological, behavioural, and neuroendocrine assessments all indicated that this model causes only moderate distress to the animals. This not only meets the need for animal ethics but also improves the quality of scientific research. Therefore, this cervical model is suitable for arteriovenous fistula research and should be used more frequently in the future.

**Abstract:**

The welfare of laboratory animals is a consistent concern for researchers. Its evaluation not only fosters ethical responsibility and addresses legal requirements, but also provides a solid basis for a high quality of research. Recently, a new cervical arteriovenous model was created in mice to understand the pathophysiology of arteriovenous fistula, which is the most commonly used access for hemodialysis. This study evaluates the distress caused by this new animal model. Ten male C57B6/J mice with cervical arteriovenous fistula were observed for 21 days. Non-invasive parameters, such as body weight, faecal corticosterone metabolites, burrowing activity, nesting activity and distress scores were evaluated at each time point. Six out of ten created arteriovenous fistula matured within the observation time as defined by an increased diameter. The body weight of all animals was reduced after surgery but recovered within five days. In addition, the distress score was significantly increased during the early time point but not at the late time point after arteriovenous fistula creation. Neither burrowing activity nor nesting behaviour were significantly reduced after surgical intervention. Moreover, faecal corticosterone metabolite concentrations did not significantly increase. Therefore, the cervical murine arteriovenous fistula model induced moderate distress in mice and revealed an appropriate maturation rate of the fistulas.

## 1. Introduction

As vascular access for hemodialysis, all academic guidelines suggest autologous arteriovenous fistula (AVF) as the first option [1,2]. Although native AVF show the lowest risk of complications, the lowest need for intervention and the best long-term patency [1], it is still the Achilles’ heel for patients [3]. After fistula creation, there are several factors that can cause fistula dysfunction, such as immaturation, thrombosis or neointimal hyperplasia [4]. In order to understand the involved mechanisms, researchers have attempted to find an effective animal model to study the underlying mechanisms.

Animal models are crucial to study physiology and pathophysiology for almost all diseases in humans and in animals as well as to develop new therapies in all fields of medicine. Appropriate animal models should strictly follow ethical standards to maximise the likelihood of obtaining the knowledge sought balanced against minimising animal discomfort. Over the past few decades, various rodent animal models have been used to study AVF maturation. For example, AVFs were created between the aorta and the inferior cava vein in a side-to-side manner [5] or by anastomosis of the common carotid artery to the jugular vein in an end-to-end manner [6]. However, none of these models mimics the anatomical characteristics of AVF in hemodialysis patients, which determines the hemodynamic profile within the fistula. This is very important because the blood flow dynamics have an important impact on AVF maturation and dysfunction [7]. Recently, researchers have created a murine AVF model in which the end of a branch of the external jugular vein was anastomosed to the side of the common carotid artery [7]. It mimics similar hemodynamic properties to classical AVF in humans, in which the anastomosis is also created in a side-to-end fashion. This improves the translation of experimental in vivo results in clinical settings. Because the new cervical model uses a small vein for AVF, the volume stress on the heart is expected to be reduced as well. However, it is not known what distress the model induces in the experimental animals.

In order to quantify the distress of animals in different experimental models, several parameters can be assessed. Body weight is a classical and essential indicator of animal distress, and all committees use body weight as an important experimental animal welfare indicator [8]. Faecal corticosterone metabolites (FCMs) reflect adrenocortical activity and are used increasingly often as a non-invasive assessment of distress [9,10]. Burrowing and nesting activity are innate behaviours in rodents. These behaviours are often affected by pain or suffering [11]. Therefore, it is possible to assess animal welfare by analysing changes in burrowing and nesting activity. This study was conducted to assess whether the cervical AVF causes a significant increase in distress for the animals and to provide objective evidence to support the use of this new animal model in future AVF studies.

## 2. Materials and Methods

### 2.1. Animals

All experiments were conducted in accordance with the European Directive (2010/63/EU). Twelve male C57BL/6 mice were used at an age of 9–15 weeks and a body weight of 20–30 g. Mice were bred in standard laboratories with a 12:12 h light-dark cycle, constant temperature (21 ± 2 °C) and humidity (60 ± 20%) and provided free access to chow and water ad libitum.

### 2.2. Experimental Design

**Figure 1:**
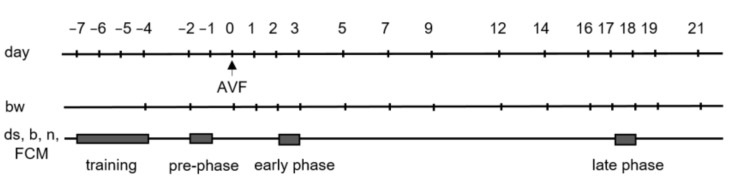
Experimental timeline for assessment of animal distress. The fistulas were created on day 0 and mice euthanized on day 21. The mice had a training phase to learn burrowing. Body weight (bw), distress score (ds), burrowing (b) as well as nesting (n) activity and faecal corticosterone me tabolites (FCMs) were evaluated as indicated.

It is known that burrowing behaviour can be affected by learning and is enhanced by social facilitation [12]. Therefore, a group training with two to four mice per cage was performed on three days in a row, starting on day −7 (shown in Figure 1). Although single housing of mice may cause distress, we decided to separate mice into single cages from day −4 because group housed male mice can show aggressive behaviour [13]. The separation also allowed us to assess burrowing activity, nesting activity and FCM concentrations for individual mice. These data were assessed from unaffected mice before AVF creation (pre-operative phase) and at various time points after AVF creation. Please note that data from the pre-operative phase served as control to data assessed after AVF creation. Faeces were collected on days −2, 2 and 17. On days −2, 2 and 17, burrowing behaviour and distress score were assessed. One day later, nesting activity was studied respectively. The time points of data assessment were described as pre-operative phase (day −2/−1), early phase (day 2/3) and late phase (day 17/18). In order to analyse continuous body weight change, the body weight was assessed on days −4, −2, 0, 1, 2, 3, 5, 7, 9, 12, 14, 16, 17, 18, 19 and 21. On day 21, the patency of the fistula was checked directly by opening the wound, and the mice were euthanized.

### 2.3. AVF Model and Tissue Harvest

AVF creation was performed as previously described [7]. In brief, mice were anaesthetised by continuous isoflurane treatment (1.5% isoflurane; 0.8 L/min N_2_O; 0.8–1.0 L/min O_2_). After subcutaneous (s.c.) injection of heparin (1 U/kg bw), the left dorsomedial branch of the external jugular vein and the ipsilateral common carotid artery were separated through ventral incision. The left sternocleidomastoid muscle was cauterized, and the carotid artery was clamped. An incision of the same diameter as the artery was made on the lateral side of the artery with micro scissors, followed by local rinse with heparin (200 U/mL). The distal end of the vein branch was ligated, cut and anastomosed to the common carotid artery in a side-to-end fashion using 10-0 Ethilon (Johnson & Johnson Medical GmbH, Norderstedt, Germany) interrupted sutures. The clamps were then released in a distal to proximal order. Confirming no active bleeding, the neck incision was closed with 5–0 Prolene (Johnson & Johnson Medical GmbH, Norderstedt, Germany). Finally, 0.5 mL of saline and 5 mg/kg bw of carprofen were injected s.c. for volume institution and pain relief, respectively. For pain relief, 1250 mg/L metamizol (Ratiopharm, Ulm, Germany) was provided daily in the drinking water after AVF creation. Mice were euthanized on day 21 by intraperitoneal (i.p.) injection of ketamine (90 mg/kg bw) and xylazine (25 mg/kg bw). To harvest the fistula vein and the contralateral dorsomedial branch of the external jugular vein as control, first 0.9% NaCl was injected intracardially in order to flush the blood out of the veins. Then, 4% formalin (Formafix, Grimm med. Logistik GmbH, Torgelow, Germany) was injected in the same way and also flushed around the veins to fixate the tissue. For histological analysis, 4-µm-thick cross-sections of both the fistula and the control vein were stained by haematoxylin-eosin (HE).

### 2.4. Body Weight and FCM Analysis

The body weight of the mice was measured four times per week with a scale (EMB 200-2, KERN & SOHN, Balingen, Germany) in the morning at 9:00–9:30 a.m. At each time point, the percentage of body weight change was determined by comparison to the body weight before AVF creation on day −4.

For analysing FCMs, all bedding with old faeces was removed, and fresh bedding was added to the cages 24 h prior to the start of faeces collection. More than 400 mg of fresh faeces were collected per mouse and dried for 4 h at 65 °C. Until further processing, the faeces was stored at −20 °C. Thereafter, 50 mg of dried faeces were extracted with 1 mL of 80% methanol for further analysis by a 5α-pregnane-3β,11β,21-triol-20-one enzyme immunoassay [10,14].

### 2.5. Assessing Burrowing and Nesting Activity

A burrowing tube (15 cm length × 6.5 cm diameter) which was filled with 200 ± 1 g food pellets (ssniff Spezialdiaeten GmbH) was placed in the left back corner of the cages 3 h before the dark phase at 4:00–4:10 pm. Mice had free access to these pellets. Nesting material was left in the cages. The weight of the food pellets (g) that remained in the tube was measured after 2 h at 6:00–6:10 pm. Then, the same tube was put back and measured on the next morning at 9:00–9:15 am and deducted from 200 g.

For evaluating nesting activity, a cotton nest-building material (5 cm square of pressed cotton batting, Zoonlab GmbH, Castrop-Rauxel, Germany) was placed in the left front of the cage 3 h before the dark phase at 4:00–4:15 pm. Pictures of the nests were taken, and nesting was scored the next morning at 9:00–09:15 a.m. This score was previously described in detail [12].

### 2.6. Distress Score Analysis

Based on the distress score sheet designed by Paster et al. [15], our working group made some modifications (for details also see Supplementary Tables S1 and S2) [16]. In brief, the scoring sheet has a total of 66 points and consists of five parts, body weight, general condition, spontaneous behaviour, flight behaviour and process-specific criteria. The distress score was assessed by only one person in a not blinded manner, according to the scoring sheet. All scores were assessed in the morning at 9:00–9:30 a.m.

### 2.7. Statistical Analysis

Data were graphed and analysed with GraphPad Prism8 (version 8.0.1, GraphPad Software Inc., San Diego, CA, USA). They were presented as single data points plus median and 95% confidence interval. Since the data describing percentage of body weight, FCM, burrowing activity, nesting score and distress score were non-parametric (as assessed by Shapiro Wilk test), one-way repeated measure ANOVA on ranks (Friedman Test) were performed by comparing each time point to the pre-operative phase (day −2 or day −1) and by correcting for multiple comparisons (Dunn’s method). Overall, statistical significance was set at *p* < 0.05.

## 3. Results

### 3.1. Mortality of Mice and Maturation of Fistulas

**Figure 2:**
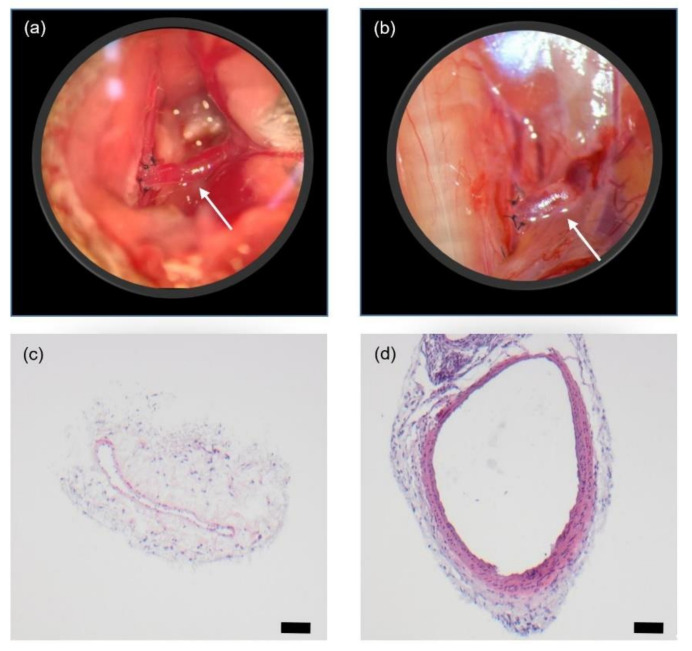
Characteristics of AVF. Morphology of AVF (white arrows) immediately after fistula creation (**a**) and on day 21 (**b**). Magnification = 40×. Haematoxylin-eosin stained cross-sections from of a control vein (**c**) and a respective fistula vein on day 21 after creation of the arteriovenous anastomosis (**d**). Scale bar = 50 µm.

One AVF was created in every mouse. Two mice were euthanized during surgical intervention due to severe bleeding, while in the other ten mice, AVF was created successfully (shown in Figure 2a). On day 21, six of ten fistulas matured sufficiently (shown in Figure 2b) as defined by patency and an increased diameter compared to the contralateral jugular vein. Four fistulas did not mature and were found occluded on day 21. Compared to control veins, HE staining of patent fistulas revealed a marked increase in both diameter and thickness of the vein wall as a sign of sufficient maturation (shown in Figure 2d).

### 3.2. Analysis of Animal Distress

**Figure 3:**
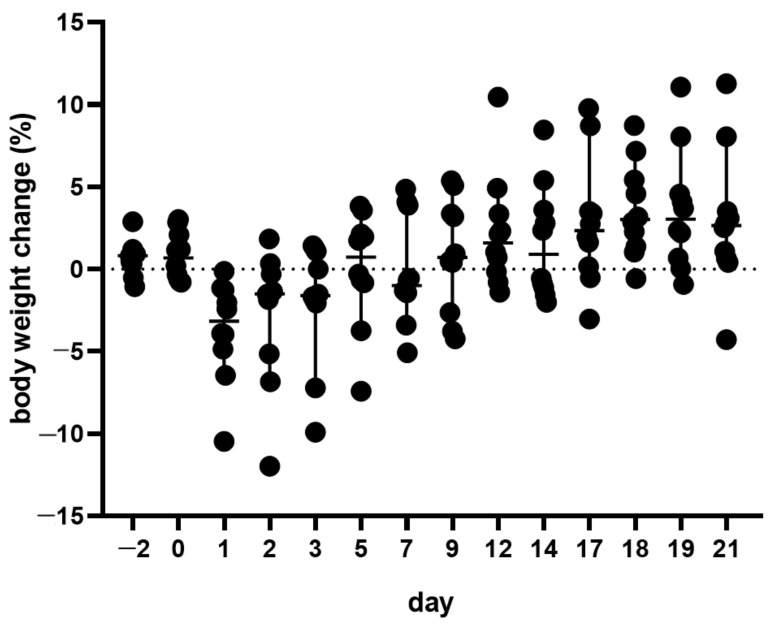
Body weight change of mice before and after creation of an AVF on day 0. Median with 95% CI. *n* = 10.

The body weight decreased after AVF creation but recovered at day 5 after surgical intervention and steadily increased until day 21. However, there was no significant difference compared to the pre-operative phase, on day −2 (shown in Figure 3). It is worth pointing out that in this early phase, nine mice lost weight in the range of 10% or less, only one mouse lost more than 10% of its body weight.

**Figure 4:**
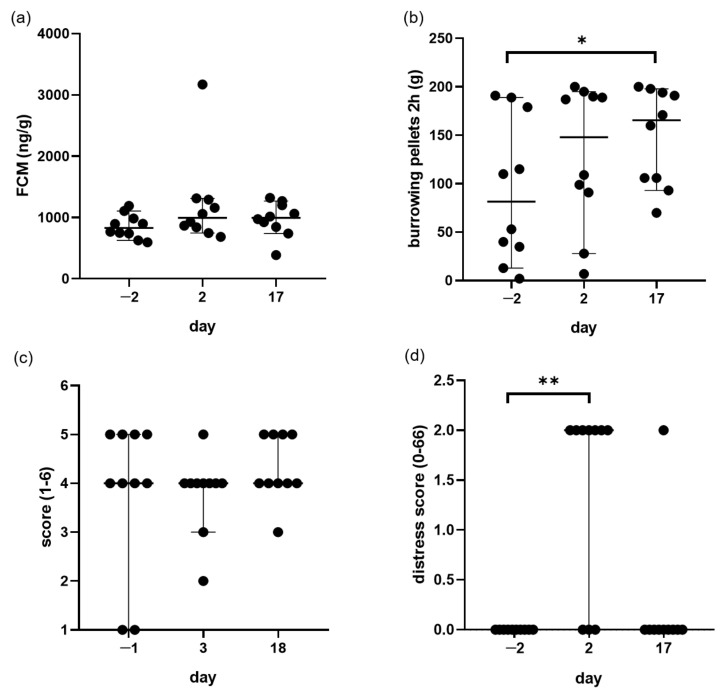
Evaluation of distress parameters during the maturation of AVF. FCM concentrations (**a**), 2-h burrowing activity (**b**), nesting activity (**c**) and the distress score (**d**) were assessed on the indicated days during pre-operative phase (day −2 or day−1), at the early time point (day 2 or day 3) or late time point (day 17 or 18) after AVF creation. Median with 95% CI. * *p* = 0.0073. ** *p* = 0.0378. *n* = 10. Each circle mean one animal.

There was a slight, but not significant (*p* = 0.1473) increase in FCM concentrations on day 2 after completion of the AVF creation, from 831/628–1106 on day −2 to 993/746–1312 ng/g (median/95% confidence interval) on day 2 (shown in Figure 4a). Notably, one mouse that lost over 10% of its body weight revealed an increase of FCMs to 3170 ng/g on day 2. In this mouse, FCM level decreased noticeably on day 17.

Analysis of burrowing activity after 2 h showed no reduction of burrowing activity during the early and late phase after AVF creation (shown in Figure 4b). Indeed, compared to day −2, the burrowing behaviour was actually significantly more intense on day 17 (*p* = 0.0073). When mice were allowed to burrow the entire night, almost all mice burrowed all pellets out of the tube. Thus, no significant difference was observed when comparing the two time points after AVF creation to the pre-operative phase (data not shown). It is noteworthy that one mouse showed a decreased burrowing activity on day 2, but that the burrowing behaviour recovered on day 17 (data not shown). This was the identical mouse, which lost more than 10% of its body weight and revealed an increased FCM concentration of 3170 ng/g on day 2.

At the different time points, all mice were similarly active in building their nests (shown in Figure 4c). The nesting scores were 4/1–5, 4/3–4 and 4/4–5 (median/95% confidence interval) on day −1, day 3 and day 18, respectively.

Notably, in the early phase, the distress of some mice increased (shown in Figure 4d). The distress scores were 0/0–0 (median/95% confidence interval) on day −2 and 2/0–2 on day 2. On day 17, the distress score returned to 0 for most mice.

## 4. Discussion

This study demonstrates that cervical AVF matures successfully and that mice after AVF experience only suffered moderate distress. Therefore, it might be stated that the cervical murine AVF model is a suitable animal model for studying vascular remodelling of an arteriovenous hemodialysis access.

C57BL/6 is the most common inbred mouse strain and has been widely used as a genetic background for studying genetically modified mice to evaluate the molecular mechanisms underlying human disorders. The purpose of the animal model presented here was to create an AVF in C57BL/6 mice to facilitate research on the molecular mechanism of AVF maturation. Although the learning curve for the challenging microsurgical creation of this cervical AVF model is long and needs continuous training, this mouse model has its own advantages compared to large animal AVF models, in which AVF creation is technically easier. For example, fistulas of this mouse model showed significant pathophysiological changes within 4 weeks [7]. This allows scientists to study fistula maturation within a fairly short period of time.

Although the vessels are much smaller in the cervical AVF model in mice compared to humans, the anatomical configuration and blood flow characteristics of the fistula are similar to AVF for hemodialysis access in humans. In addition to anatomical consistency with the clinical condition, this animal model also has a similar clinical success rate. In this study, 40% of the fistulas did not mature and were occluded three weeks after the operation. This is in line with the description of Wang et al. [7]. Since the patency rate is lower than 100%, this animal model also allows researchers to assess therapeutic approaches for an improvement of fistula maturation. In addition, the immature fistulas provide an opportunity to study the mechanism of AVF immaturity by means of histological, immunohistochemical or biomolecular analysis. Compared to native veins, patent fistulas showed increased diameter and thicker vessel walls. These characteristics are also found in human fistulas. In humans, neointimal hyperplasia is a common pathological manifestation during fistula maturation [4]. HE staining revealed markedly neointimal hyperplasia compared to contralateral native veins. These findings suggest that this model can be used to study the mechanisms of fistula maturation as well as different treatment options. It is notable that all successfully created AVF mice survived to the end of experiment. No mouse died due to complications, such as heart failure, which is a main cause of distress after AVF creation [17,18]. The novel model uses a smaller diameter vein, a branch of the external jugular vein, as a venous fistula. This may reduce the cardiac load and the risk of heart failure, which might explain the moderate level of distress. Thus, this animal model might reduce the number of animals needed for pursuing research in this field without causing severe distress to animals.

Improvement in animal welfare is important for pursuing basic as well as preclinical research [19]. In order to quantify the distress of this animal model, body weight, FCMs, burrowing activity and nesting behaviour as well as distress score were chosen as distress indicators.

Body weight is the most common parameter for assessing distress. As early as 1985, it had been used as a key indicator to evaluate animal distress [20]. Because weighing is non-invasive and objective, almost daily weight had been done to see the dynamics of weight change. Before operation, without AVF stressor, all mice showed good health status and progressive weight gain. Continuous observation of body weight revealed that there was a weight loss in the early phase, which could be explained by surgery. Surgery and anesthesia are both particularly potent stressors [21,22], and thus post-surgical body weight reduction is common in mice [16,23,24,25]. However, as the experiment progressed, the mice recovered and gained stable weight over the next 3 weeks. Therefore, the early phase and the late experimental phase were chosen as time points to assess the distress using additional parameters. As seen in other studies on distress and weight change [23], when mice suffered severe distress, the body weight of the experimental animals started to decrease progressively. Although the surgery itself had an impact on the welfare of the mice, the noticeable increase in body weight at the late phase indicated that the mice were in good condition, and that the AVF itself did not cause severe distress to the animals.

It is known that FCM levels positively correlate with distress [9,10]. The advantage of faeces samples over blood samples is that they can be easily collected without additionally stressing the animals [26]. In the present research, consistent with other experimental results [24,25,27], there was a moderate increase in FCM level after the respective intervention. However, there was no statistically significant difference compared to the pre- operative phase. After AVF creation, FCM levels did not increase over the course of time, which indicates that the cervical fistula did not significantly increase distress in mice. It is noteworthy that one mouse had a particularly high FCM level in the early phase, corresponding to a loss of more than 10% body weight. Compared to other mice, this mouse had more intraoperative loss of blood, which may have contributed to its significant weight loss and stress [28]. This observation also indicates that the performance of the surgeon has the biggest effect on the animal’s well- being.

Since not every type of stressor may be reflected in corticosterone levels [29], behavioural parameters were also detected in our research to assess animal distress. Numerous studies have demonstrated that burrowing behaviour and nesting activity are reduced by distress [12,30,31]. Similar to body weight, burrowing activity can also be assessed objectively, which is easy to perform. In order to induce minimal distress and unnecessary effects on the mice, burrowing and nesting behaviour were tested inside the home cage [32]. During fistula maturation, there was no decrease in burrowing activity observed. Mice actually burrowed more pellets in late phase. Since some mice pulled the nesting material into the burrowing tube, burrowing and nesting activity were assessed on different days. Unlike body weight, FCMs and burrowing activity, the nesting score is a subjective score; however, deficits of nest building also reflect a decline in animal welfare and impaired general condition [30]. In agreement with other studies [24,25,27], most mice were highly motivated to build nests in this study, but no significant difference between each time point was observed.

In addition, a distress score sheet was used to evaluate AVF-bearing mice. Our work group has used this distress score sheet several times to successfully analyse distress in experimental mice [16,23,24,25,27]. All mice showed low distress scores, no more than 2 out of 66 theoretically possible points. Consistent with the results of other parameters, the distress also reflected that this animal model only moderately increased distress. However, we want to mention that the distress score has a similar limitation to the nesting score in that it is not measured objectively but in a subjective manner.

We also want to describe other limitations of this study. Since the behavioural analysis of burrowing and nesting requires the mice to be housed separately, this separation may cause distress and might influence our analysis. However, we did not observe any obvious signs of distress on single housed mice before surgical intervention. Another limitation is the small number of mice tested in this experiment. This might prevent observing statistically significant differences between different time points. Since this is a new animal model, only a small number of animals was used to test the feasibility and validity of this animal model. However, distress of additional animals should be evaluated to further validate the feasibility and safety of this model.

## 5. Conclusions

The presented cervical AVF model matures successfully. The analysis of multiple distress parameters, such as body weight, FCMs, burrowing activity, nesting activity and a distress score, demonstrated that this model in mice can be established without causing severe distress. Therefore, it is a suitable model to study the pathophysiology of vascular remodeling in AVF. It should be noted that the mice in this study did not suffer renal insufficiency or uraemia, which are key factors affecting fistula maturation and neointima formation [33]. Therefore, kidney dysfunction should be added to this model, and the distress caused by renal insufficiency plus AVF should be carefully evaluated.

## Data Availability

The data presented in this study are available on request from the corresponding author. The data are not publicly available to preserve privacy of the data.

## Supplementary Materials

The following are available online at https://doi.org/10.3390/ani9040145, Table S1: Distress score on mice [16]; Table S2: Consequences according to distress score [16].
